# An Account of an Experiment Lately Made at Florence in a Case of Hydrophobia

**Published:** 1788

**Authors:** J. Fabbroni

**Affiliations:** Assistant Director of the Cabinet of Natural History of his Royal Highess the Grand Duke of Tuscany, and Secretary of the Royal Academy of Agriculture at Florence


					IX.
An Account of an Experiment lately made at
Florence in a cafe of Hydrophobia.
Communi-
cated by Mr. J. Fabbroni, Affiftant Director of
the Cabinet of Natural Hijiory of his Royal
Highnefs the Grand Duke of Tufcany, and Sec-
retary of the Royal Academy of Agriculture at
Florence, in a Letter to Sir Jofeph Banks,
Bart. P. R. S. and by him to Dr. Simmons.
ENURING the laft fummer we had a great
r number of mad dogs in the neighbour-
hood of this city, and in the city itfelf, Seve-
ral
C 70 ]
ral "pcrfons, who were bit by them, died of
hydrophobia j and their bodies were examined
after death, but without affording any infor-
mation relative to the difeafe. The vilcera
were uniformly healthy, except in one fubjeift in
which the lungs were found adhering to the
pleura; but, in all of them, the brain was
obferve.d to be more loaded with blood than
ufual.
In one cafe, an experiment, which the phyfi-
cians here have long had an idea of making *,
and from which they were not without fomc
hopes, was tried. It was indeed fomewhat
bold, but in the horrid and hopelefs flate to
which the unhappy patients in iuch cafes are
reduced, every thing feems to be allowable;
and the perfon on whom the experiment was
tried appeared to be fo near his end, that it:
was thought he could not poffibly furvive morq
than an hour.
In this cafe, a viper was applied to each of"
the patient's legs, and at the very inftant of
the bite the fymptoms feemcd to increafe in
'* We..formerly had occafion to mention, that M. de Mat-
thiis, a Neapolitan furgeon, from the event of an experiment
on a dog, had recommended the bite of the viper, as a re-
medy in hydrophobia. See vol. v. p. 220.?Editor.
violence;
L ??? 3
violence ; but this was only momentary, as he
immediately became more calm and colle&ed*
gave an account of his relations, afked for
Something to drink, and even drank; but died
within half an hour.
This experiment did not feem to be at all
concluflve either for or againft; but it occa-
fioned fo much popular clamour, that I think it
will hardly be repeated here, at leaft on a
human fubjedt*.
Florence,
Nov. 2 0} i 7 S 7 ^
It certainly deferves to be recorded, that fuch an expe-
riment, as the one here related, has been made in the hydro-
phobia ; but it Would feern, that at the time the-vipers were
applied, nothing fatisfa&ory could be cxpe6led from this ov
sny other experiment.?Editqs.,

				

